# Perceptions and Use of Automated Hospital Outcome Data by EMS Providers: A Pilot Study

**DOI:** 10.5811/westjem.21175

**Published:** 2024-10-01

**Authors:** Michael Kaduce, Antonio Fernandez, Scott Bourn, Dustin Calhoun, Jefferson Williams, Mallory DeLuca, Heidi Abraham, Kevin Uhl, Brian Bregenzer, Baxter Larmon, Remle P. Crowe, Alison Treichel, J. Brent Myers

**Affiliations:** * Falck Health Institute, Orange, California; † ESO, Austin, Texas; ‡ University of Cincinnati, Cincinnati, Ohio; § Wake County Emergency Medical Services, Wake County, North Carolina; ∥ Austin-Travis County Emergency Medical Services, Austin, Texas; ¶ Cincinnati Fire Department, Cincinnati, Ohio; # University of California Los Angeles, David Geffen School of Medicine, Los Angeles, California

## Abstract

**Background:**

Our primary objective evaluated the perception of emergency medical service (EMS) providers’ review of automated hospital outcome data. Secondarily, we assessed participation in outcome review as a means of microlearning to obtain continuing education (CE).

**Methods:**

From October–December 2023, three high-volume EMS systems participated in a three-part intervention with results evaluated using a mixed-methods approach. First, EMS providers (emergency medical technicians and paramedics) were invited, via their electronic health record (EHR), to complete a presurvey evaluating their perceptions of reviewing outcomes. Then, EMS providers were notified about the opportunity to earn CE via a microlearning intervention, offering Commission on Accreditation for Pre-Hospital Continuing Education (CAPCE)-approved CE hours for completion of outcome reviews and associated learning modules. Finally, EMS providers were invited to complete a post-survey mirroring the pre-survey. Qualitative analyses identified themes among open-ended responses. Quantitative analyses examined perceptions between pre- and post- surveys.

**Results:**

Of 843 providers contacted, 217 responded to the pre-survey (25.7%). The most endorsed rationale for reviewing outcomes included improving clinical knowledge (95%), improving patient care (94%), and knowing whether care made a difference (93%). Nearly all (91%) reported being more likely to review outcomes if CE were awarded. Among the 67 who completed the open-ended items, the three dominant themes included enhance personal confidence and competence (43%); acquire personal knowledge (39%); and operations (21%). Of 211 providers who participated in the intervention, 56 (27%) were awarded CE. A total of 152 providers responded to the post-survey, and the percentage who agreed that reviewing outcomes improves job satisfaction rose from 89% to 95% between pre- and post-surveys (*P* = 0.05).

**Conclusion:**

EMS providers supported the personal and professional development and patient care improvement of reviewing patients’ outcomes with associated CE. Further study is warranted to evaluate the generalizability of these findings and the best user experience.

Population Health Research Capsule What do we already know about this issue?

*EMS providers receive limited formal clinical feedback or microlearning continuing education (CE) following the treatment and transfer of their patients.*

What was the research question?

*What are the perceptions of EMS providers’ review of automated hospital outcome data and associated CE credit?*

What was the major finding of the study?

*Following outcome review and CE opportunities, surveys demonstrated job satisfaction rose from 89% to 95% (P = 0.05).*

How does this improve population health?

*EMS review of patient outcomes improves job satisfaction and clinical knowledge, thus providing a means for continued competency of a highly trained EMS workforce.*

## BACKGROUND

Emergency medical services (EMS) providers provide the majority of prehospital medical care in the United States and serve as a crucial component of the nation’s healthcare delivery system. For EMS providers to maintain their license, every state has unique continuing education (CE) requirements, typically in the fashion of required hours, for licensure renewal. The National Registry of Emergency Medical Technicians also maintains an hour and topic requirement for recertification. This hour model has been used to ensure the continual competency of EMS providers,^1^ as it has been historically accepted that competency can be maintained through CE hour requirements despite a lack of empirical evidence.^2^

In continuing medical education (CME), a broader concept of continued professional development (CPD), is emerging. Continued professional development includes education focused on problem identification and solution development, allowing the healthcare professional to tailor the learning process to their individual needs.^3^ The process of CPD recognizes a one-size-fits-all approach, which is not specific enough for each learner. The Institute of Medicine recommends a CPD system that includes patient-centered care, interprofessional teamwork, quality improvement application, and clinical outcome data utilization for individual, team, and institutional assessment.^2^ ^,^ ^4^ Feedback has also been demonstrated to improve system performance and patient outcomes.^5^ Very public voices, including the EMS Agenda 2050, have included calls for EMS systems and providers to receive feedback, including patient outcomes, in real time, as a means for continuous quality improvement, thus moving toward a CPD approach to competency.^6^ The National Association of EMS Physicians has also called for continual monitoring of airway performance data and its use in the continued credentialing process and quality management activities with large-scale bidirectional information shared between EMS and receiving facilities in their position paper on airway management and training.^7^

Despite this desired transition to CPD, providing patient-specific outcomes to EMS providers has long been a challenge. Bidirectional data sharing between EMS and hospitals has raised concerns about patient privacy and technological challenges^8^ ^,^ ^9^ Fortunately, this trend is improving, based on the provision of outcome elements as a part of the National Emergency Medical Services Information System dataset and the clarification that such data-sharing is consistent with the Health Insurance Portability and Accountability Act guidelines.^10^ ^–^ ^12^ When surveyed, EMS providers reported a desire for patient-specific outcomes. They even reported using informal networks or going around the system to obtain patient outcome information, assisting them to develop clinical skills.^7^ This lack of insight, specifically centered around patient outcomes and EMS provider diagnosis accuracy, has been reported to impact provider mental health.^13^

The EMS provider is interested in including patient outcome data as a means of professional development similar to their colleagues in medicine.^14^ ^,^ ^15^ Physicians’ continued medical education has included electronic health records (EHR) to assess the quality of care and has been used to suggest areas for improvement through the use of CME.^16^ Similarly, medical databases have found ways to help physicians receive CME with routine clinical questions and problem-solving in their daily practice.^17^ Graduate medical education also envisions a system in which patient health records and outcome data can be incorporated into the curriculum. This framework includes mentors or instructors using the outcome data to assess, supervise, and teach, creating a mature, professional community where everyone receives and provides feedback.^18^ The Accreditation Council for Graduate Medical Education similarly requires programs to connect resident-physician education to patient outcomes.^19^

Using patient outcome data as feedback continues to be called for and, on a small scale, has been demonstrated to be an effective part of CPD programs for EMS providers. In fact, the EHR serves as a valuable resource for CPD in providing patient-specific outcomes.^20^ When evaluating CE in pediatric emergencies, brief and frequent CE programs are recommended as a means to provide repetition with immediate feedback and error correction.^21^ While feedback following a call is outside the proposed theoretical framework for clinical judgment in EMS, it has been noted as important in the development of EMS providers and allows for improvement in performance.^22^ Post-resuscitation provider feedback for patients who have suffered cardiac arrest and heart attacks has led to improvements in time and treatment.^23^ ^,^ ^24^ Sammuel and colleagues conducted a scoping review of the effects of CPD on healthcare professionals’ performance and patient outcomes and were also able to demonstrate changes in providers’ behavior and patient outcomes.^25^

Despite calls from leading national EMS organizations and other healthcare professions to incorporate patient outcomes into CE, little is known about EMS providers’ perceptions of automated patient outcome data nor its use as microlearning to obtain continuing education. Our primary objective in this study was to evaluate EMS providers’ perceptions of the utility of automated hospital outcome data for professional development. Secondarily, we evaluated their participation in outcome review as a means of microlearning to obtain CE.

## METHODS

### Study Design and Population

In this mixed-methods study we ued quantitative and qualitative methods to understand EMS provider perceptions regarding the use of an automated system enabling them to obtain Commission on Accreditation for Pre-Hospital Continuing Education (CAPCE)-approved CE by reviewing patient outcomes and completing associated learning modules. The CAPCE is an accrediting body charged with the review and accreditation of EMS CE.^26^ The study was conducted from October–December 2023 in three high-volume urban EMS systems. The EMS providers were certified at the emergency medical technician through the paramedic level and were continuously provided 100% of their required CE hours through their employer.

At the beginning of the study, EMS providers in each system received a prompt inviting them to anonymously participate in this pilot study when they logged into their EHR (ESO, Austin TX). Each participating agency uses a system that allows EMS providers to automatically receive outcome data from the hospital, specific to patients they encountered in the prehospital setting. All CE activities were completed in a single online learning platform.

For those who agreed to participate in the first phase of the pilot study, participants were asked to voluntarily complete a pre-survey. They were also informed that they would be eligible to receive CE hours, approved by CAPCE, upon completion of outcome reviews, associated videos, and outcome review assessments. A prompt to review outcomes as part of this study was received by participants when they logged into the EHR and navigated to the outcome review section. The CE phase was open for at least two weeks. This was followed by another invitation in the EHR to complete the post-survey that mirrored the pre-survey. Participants were required to specifically opt into each survey and the CE phase of the study.

### CAPCE-Approved Continuing Education

During the CE phase, participants were provided a link from the outcome review page that directed them to login to the learning platform. Two prerequisite videos provided background information and described the types of outcome information available: International Classification of Diseases diagnosis codes and Centers for Medicare and Medicaid Services disposition (eg, discharge to home, discharge to skill nursing, transfer, death, etc). Upon completion of these prerequisites, participants were able to review their patient-specific outcomes and answer a series of questions regarding each review.

### Data Analysis

We used quantitative analyses to examine perceptions between pre- and post-surveys using chi-square tests or Fisher exact tests, as appropriate. Likert-scale response options (agree vs disagree) and demographics (Level: paramedic vs other; Role: patient care provider vs other; years of experience: 0–4 years, 5–10 years, 11–20 years, ≥21 years; previous frequency of outcomes review: very frequently/frequently vs occasionally/ rarely/never) were collapsed, as needed and where appropriate, due to cell size. Participation in the CAPCE CE phase of the study was also quantified. Data is reported as percentages and frequencies. *P*-values of statistical tests are also included. We performed quantitative analysis using STATA MP version 18.0 (StataCorp LLC; College Station, TX).

Qualitative analyses identified themes among open-ended responses. We used conventional content analysis as described by Hsieh and Shannon (2005). An inductive approach was used to extract meaning and themes from responses. All codes were generated from the content directly without a priori themes. Once codes were identified, we used a deductive approach to determine the frequency and distribution of themes as well as any relationships that existed between themes and respondent characteristics. Qualitative analysis was assisted using the QDA Miner Lite 3.0 (Provalis Research; Montreal, Quebec) software package.

This study was deemed exempt by the University of California Los Angeles Institutional Review Board.

## RESULTS

During the study period, 843 EMS providers from the participating agencies logged into the EHR and were therefore eligible to participate. A total of 217 (25.7%) EMS providers anonymously responded to the pre-survey while on duty, and 152 (18.0%) anonymously responded to the post-survey while on duty. Demographics were similar among those that responded to the pre- and post-survey (Table 1).

**Table 1.** Participant characteristics.

% (n)	Pre-survey (N = 217)	Post-survey (N = 152)
Certification level		
Emergency medical technician (EMT)	14.8% (32)	11.2% (17)
Advanced emergency medical technician (AEMT)	2.3% (5)	3.3% (5)
Paramedic	77.9% (169)	82.9% (126)
Other	1.8% (4)	0.0% (0)
None	3.2% (7)	2.6% (4)
Role within the organization		
Patient care professional	61.2% (131)	71.3% (107)
First-line supervisor	20.6% (44)	18.0% (27)
Administrator/manager	4.7% (10)	1.3% (2)
Preceptor	3.7% (8)	3.3% (5)
Educator	0.9% (2)	0.7% (1)
Other	8.9% (19)	5.3% (8)
Years of experience (4 categories)		
0–4 years	18.1% (39)	18.4% (28)
5–10 years	28.2% (61)	28.9% (44)
11–20 years	31.5% (68)	28.9% (44)
≥21 years	22.2% (48)	23.7% (36)
Frequency of patient outcomes review		
Very frequently (more than once per week)	38.8% (83)	36.2% (55)
Frequently (about once per week)	32.2% (69)	33.6% (51)
Occasionally (about once or twice per month)	19.2% (41)	25.7% (39)
Rarely (about once or twice per year)	3.3% (7)	2.0% (3)
Very rarely (less than once per year)	2.8% (6)	2.6% (4)
Never (I have never viewed an outcome)	3.7% (8)	0.0% (0)

Overall, responses were similar when comparing those who responded to the pre-survey and those who responded to the post-survey (Table 2). Notably, 89% in the pre-survey vs 95% in the post-survey indicated agreement that outcomes review enhanced job satisfaction (*P* = 0.05). When evaluating only those with a paramedic certification, we saw a statistically significant increase (*P* = 0.03) in the percentage of those indicating they review outcomes to improve clinical knowledge when comparing pre-survey respondents to post-survey respondents (94% vs 99%, respectively). We also noted that pre- and post-survey differences with respect to improvements in job satisfaction remained significant when only evaluating paramedics. No statistically significant difference was noted among pre-and post-survey respondents of those with other certification levels (Table S1). When examining years of EMS experience or role in the EMS system, responses between pre- and post-survey respondents were similar regardless of how many years the individual had worked in EMS (Table S2).

**Table 2.** Pre-survey and post-survey overall responses.

	Pre-survey		Post-survey		
% (n)	Disagree	Agree	Disagree	Agree	*P*-value
I review outcomes to improve the care I provide to patients.	6.0% (12)	94.0% (188)	4.1% (6)	95.9% (141)	0.43
I review outcomes to improve my clinical knowledge.	5.5% (11)	94.5% (188)	2.1% (3)	97.9% (140)	0.17
I review outcomes to know whether my care made a difference.	7.0% (14)	93.0% (186)	4.1% (6)	95.9% (140)	0.26
I review outcomes to know whether I provided the right care.	6.9% (14)	93.1% (188)	2.8% (4)	97.2% (141)	0.09
I review outcomes to obtain closure on patient encounters.	14.0% (28)	86.0% (172)	8.9% (13)	91.1% (133)	0.15
Reviewing hospital outcome data through the patient outcomes feature helps improve my job satisfaction.	11.3% (24)	88.7% (188)	5.3% (8)	94.7% (142)	0.05
Reviewing hospital outcome data through the patient outcomes feature helps improve my clinical knowledge.	6.8% (14)	93.2% (192)	6.9% (10)	93.1% (135)	0.97
If I were provided 15 minutes of approved continuing education credit for each patient outcome I reviewed, I would be more likely to review my patient outcomes in the patient outcomes feature.	8.1% (17)	91.9% (193)	9.3% (14)	90.7% (136)	0.68

There were two significant differences when comparing those who responded to the pre-survey and those who responded to the post-survey when stratified by historical frequency of outcomes review. Among those who indicated that they review patient outcomes frequently or very frequently, we saw a significantly higher percentage of post-survey respondents indicating that they reviewed patient outcomes to obtain closure (90% vs 98%, *P* = 0.01, respectively) and that reviewing patient outcomes improves job satisfaction (92% vs 99%, *P* = 0.02, respectively) (Table S3).

### CAPCE-Approved Continuing Education

**Figure 1.**
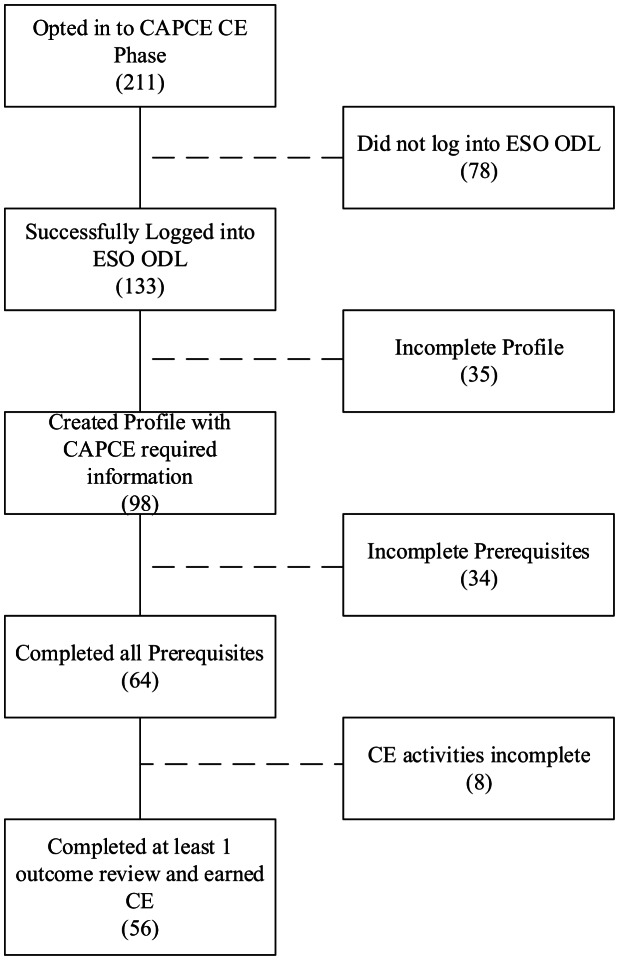
CAPCE CE optional participation. *CAPCE*, Commission on Accreditation for Pre-Hospital Continuing Education; *CE*, continuing education.

A total of 211 individuals from the three participating EMS systems opted in to the CAPCE CE phase of this pilot study. Among those, 63% (133/211) successfully logged into the ESO learning platform. Of those who successfully logged in, 74% (98/133) completed their required profile. The prerequisites were completed by 70% (64/98) of those with a completed profile, and 88% (56/64) of those completed at least one CE activity. Among those who completed any CE, 88% (35/64) completed more than one CE activity and less than 10% (4/64) completed all available CE activities (Figure 1). A total of 287 outcomes were reviewed, and responses following the outcome review activity are listed in Table 3.

**Table 3.** Continuing education activity review questions and responses.

All continuing education reviews	
Goals of your outcome review (select all that apply):	
Obtain continuing education credit for licensure renewal.	22% (224)
Increase my knowledge regarding the clinical condition of the patient.	25% (247)
Determine whether my assessment and treatments were appropriate.	26% (258)
Determine whether my field impression aligns with the hospital diagnosis.	27% (267)
Based on this review:	
I would provide the same treatment.	84% (242)
I need more information.	6% (17)
I would modify my treatment.	10% (28)
Indicate how strongly you agree or disagree with the following: My use of outcomes reinforces or increases my clinical knowledge.
4 – strongly agree	52% (149)
3 – agree	46% (132)
2 – disagree	0% (1)
1 – strongly disagree	2% (5)
Indicate how strongly you agree or disagree with the following: My use of outcomes improves the quality of care I provide to my patients.
4 – strongly agree	50% (142)
3 – agree	49% (139)
2 – disagree	1% (3)
1 – strongly disagree	1% (2)
Indicate how strongly you agree or disagree with the following: If I were provided additional online continuing education opportunities related to this patient outcome, I would complete the training.
4 – strongly agree	44% (127)
3 – agree	55% (156)
2 – disagree	0% (1)
1 – strongly disagree	1% (2)

### Qualitative Analysis

There were 72 pre-survey participants who answered the free-text question that asked, “Please describe any other reasons you review outcomes.” Free-text responses were uploaded into QDA Data Miner for analysis. All coding was performed by a single researcher (SB) and reviewed by AF and MK. Most initial codes related to reasons respondents reviewed the outcomes or benefits gained by doing so. The most common code identified was *improve understanding of patients I’ve seen,* which was identified 14 times (13% of all codes). The second most identified code was a*ffirming (that my diagnosis and treatment were correct),* identified eight times (7% of all codes). We excluded from further analysis 10 respondents whose only text content was coded as “do not review outcomes” or “wish list” (a code for entries that described features they wanted to see in the future).

We grouped the codes into themes organized by common attributes. These initial codes were organized by beneficiary of patient outcome review CE: the respondent; the patient encounter; and the EMS operation (Figure S1). Notably, *unknown,* a group created for codes that didn’t seem to fit any of the identified themes, comprised 17% of all codes. As a result, we performed a second thematic analysis in an attempt to integrate “unknown” codes and identify higher level meanings behind the codes. The resulting themes represented broader personal, operational, and system-related benefits to reviewing patient outcomes. These revised themes and their associated codes were reviewed by AF and MK with minor revisions recommended and integrated.

The final themes and code groupings can be found in Figure S2. We believe these codes and themes accurately reflect survey responses, and we used them for analysis of the post-survey, free-text question using the same method. Table 4 provides an overview of the themes, dominant codes, and key quotes for pre- and post-survey groups. In addition, we compared thematic frequency reports between the entire survey group and various demographic groups by respondent level, experience, and frequency of outcome review. These results can be found in Table S1–S4.

**Table 4.** Overview of themes, codes, and key quotes from the pre- and post-survey.

Theme	Description	Dominant codes pre-survey	Dominant codes post-survey
Acquire personal knowledge	Codes that reflect learning about clinical presentations, medical knowledge, and hospital related to patients seen.	• Improve my understanding of patients I’ve seen • Further clinical knowledge	• Improve my understanding of patients I’ve seen
	Key quote: *To follow up on patient encounters and understand the care the patient later received*		
Enhance personal confidence and competence	Codes that affirm that EMS diagnosis and care were appropriate, reflect on the impact of EMS care on patient outcome, and contribute to self QA/QI.	• Affirming • Self QA/QI	• Affirming

	Key quote: *I review charts to review my findings and QA/QI myself for future patients*		
Improve personal clinical practice	Codes that reflect improvement in personal clinical practice including informing future diagnosis and treatment of recurring patients.	• Future betterment • Inform future differential diagnosis	• Inform future visits to same patient • Future betterment to improve care
	Key quote: *Helps prioritize follow-ups and what resources should be brought to bear for our clients*		
Operations	Codes reflecting impact on operational tasks including documentation, follow-ups services and training.	• It’s my job • Research billing inquiry	• Training/reinforcement for trainees • Inform follow-up post discharge
	Key quote: *I review outcome data with…employees …it helps us learn together, building the educational safety among our group*		
Support system-wide QI	Codes reflecting collaboration, culture of learning and education, and organization wide QA/QI.	• Organization-wide compares crew medical decision making, protocol utilization to patient diagnosis and outcome • System QA/QI	• System QA/QI • Create a culture of learning and education
	Key quote: *It helps create a culture in which learning and education are the focus, not errors or missteps*		

*EMS*, emergency medical services; *QA*, quality assurance; *QI*, quality improvement.

**Figure 2.**
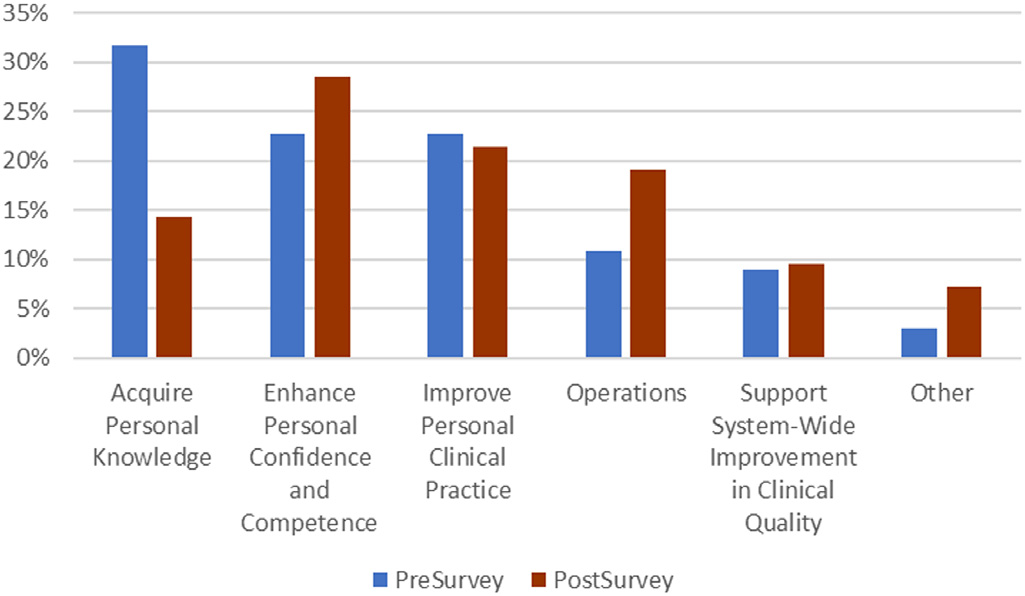
Comparison of themes pre- to post-survey.

There were shifts in the perceived value of patient outcome review between the pre- and post-survey periods and across individuals with different backgrounds and experience (Figure 2). Those respondents who indicated they did not provide direct patient care placed the most value on operations and systemwide quality. For the remainder of respondents, pre-survey results found that the predominant value identified was related to *acquiring personal knowledge,* with *enhancing personal confidence and competence,* and *improving personal clinical practice* following—nearly 10% lower. During the post-survey, *acquiring personal knowledge* fell to the fourth position, with *enhancing personal confidence and competence* the predominant theme, followed by *improving personal clinical practice* and *operations.* In the post-survey population, *acquiring personal knowledge* remained valued by EMTs and individuals with 0–4 years of experience but was not valued at all in those with ≥21 years of experience.

## DISCUSSION

### Enhance Personal Confidence/Competence and Improve Patient Care

When surveyed anonymously, EMS providers favored viewing their patient outcomes. Qualitative analysis of pre-survey results revealed that the majority of certified personnel felt the primary benefit would be increases in personal knowledge; this attitude was particularly prevalent for EMTs and individuals who had limited experience. In post-survey results, providers found that reviewing patient outcomes was more relevant to gaining confidence and competence rather than knowledge acquisition. McGuire and colleagues evaluated feedback requests received from EMS providers and found the most common request was for the final diagnosis and outcome/disposition.^28^ This enhancement is commonly reflected in EMS providers who assess and care for their patients but are limited to only the beginning of the patient’s experience, not the final outcome. Providing them with the outcome of their patient allows them to either increase confidence in their assessment and diagnosis or participate in an opportunity for continued learning. This finding represents the workforce’s desire for an outcome-centric continued competence model over traditional forms of CE.

Providers also perceived improved patient care by viewing their hospital outcome data, which previous work has demonstrated is beneficial to the learning process and can improve patient outcomes. The qualitative review of post-survey results revealed an increase in the emphasis on enhancing personal confidence and competence and improving personal clinical practice, suggesting that providers see the potential for this education to improve their clinical practice rather than just providing knowledge. Post-resuscitation feedback has also been demonstrated to improve the quality of Advanced Life Support, specifically in survival until hospital discharge and favorable neurological outcomes and is why real-life, post-resuscitation feedback is recommended.^23^ Time to treatment in heart attack victims has also been shown to decrease following the implementation of data feedback to EMS providers, even in a system that was already achieving internationally established goals.^24^ Sammuel and colleagues conducted a scoping review of the effects of CPD on health professionals’ performance and patient outcomes and demonstrated changes in providers’ behavior and patient outcomes.^25^ Survey results published by Pollard and Black found that after receiving patient outcome data, most EMS providers reflected on the call and did further reading.^29^

### EMS Provider Job Satisfaction

Study participants felt that learning their patient-specific hospital outcomes improved job satisfaction. Similar results have demonstrated that when asked, EMS providers anticipate patient outcome feedback benefiting their well-being and work engagement.^14^ Providing patient outcomes helps bring EMS providers further into the healthcare continuum, and doing so reduces the causes of burnout for them.^30^ This is an important finding as agencies face a workforce shortage and the US sees fewer and fewer EMTs and paramedics.^31^

### Microlearning

The CAPCE-approved CE was presented to EMS providers in the form of microlearning. Microlearning consists of small doses of content in the form of lesson modules or short-term activities. This method allows learners to control all aspects of their learning, including the time in which they review, the pace, and the method by which they complete the activity. Providers reported being more likely to complete the review if they were provided with a means to use it to complete certification renewal; thus, if an agency provides patient outcomes to providers, it is best to include it as a means for certification renewal.

This patient outcome-specific education describes a new method of CE for EMS providers and aligns with the goals of CPD. Additionally, patient outcome-led education has been demonstrated to improve provider competency and improve patient outcomes. More study is needed on a wider scale to determine whether this type of education delivery ensures more competency than an hour-based model. This type of learning may also be appealing from an operational perspective, providing flexibility with respect to scheduling educational offerings and reducing the time that EMS providers are required to spend in a classroom.

## LIMITATIONS

The sample size is specific to these three agencies (convenience sample) and those who chose to complete the survey; as such it may not be generalizable to the entire EMS workforce. The window for completing the survey was open for two weeks; thus, there may have been some providers who did not work in that period and could not complete the survey. Additionally, the request to complete the survey occurred during operational hours; so, participation may have been limited due to other, more urgent tasks. By its nature, qualitative analysis is heavily dependent on the background and skills of the researcher and may be influenced by personal biases.

A low proportion of providers who began the CE process actually gained CE credits, indicating the steps or process may be too difficult or cumbersome. It is also possible that participants did not need the CE credits, as each of the participating agencies provides 100% of the required hours. Additional research is needed to determine how to best integrate this learning method with existing work patterns and CE programs.

## CONCLUSION

Emergency medical services providers supported the personal and professional development and patient care improvement value of reviewing patient outcomes, including microlearning activities. Participation in the required activities to obtain continuing education was low. However, subjects who did participate demonstrated a shift in perceived value from mere acquisition of knowledge to development of improved personal and systemwide clinical practice. Further study is warranted to evaluate the generalizability of these findings and the best user experience to facilitate the completion of CE.

## Supplementary Information





## References

[1] Terry M Powell J Gilmore WS et al . Deriving national continued competency priorities for emergency medical services clinicians. Prehosp Emerg Care. 2023;27(4):439–48. 36066437 10.1080/10903127.2022.2120934

[2] Studnek JR Fernandez AR Margolis GS . Assessing continued cognitive competence among rural emergency medical technicians. Prehosp Emerg Care. 2009;13(3):357–63. 19499473 10.1080/10903120902935355

[3] DeSilets LD . The Institute of Medicine’s redesigning continuing education in the health professions. J Contin Educ Nurs. 2010;41(8):340–1. 20666352 10.3928/00220124-20100726-02

[4] Balmer JT . The transformation of continuing medical education (CME) in the United States. Adv Med Educ Pract. 2013;4:171–82. 24101887 10.2147/AMEP.S35087 PMC3791543

[5] Ivers N Jamtvedt G Flottorp S et al . Audit and feedback: effects on professional practice and healthcare outcomes. Cochrane Database Syst Rev. 2012;(6):CD000259. 22696318 10.1002/14651858.CD000259.pub3 PMC11338587

[6] (2019). EMS Agenda 2050 Technical Expert Panel, National Highway Traffic Safety Administration, Department of Tranportation. EMS Agenda 2050: a people-centered vision for the future of emergency medical services, report No. DOT HS 812 664. Washington, DC: National Highway Traffic Safety Administration.

[7] Dorsett M Panchal AR Stephens C et al . Prehospital airway management training and education: an NAEMSP position statement and resource document. Prehosp Emerg Care. 2022;26(sup1):3–13. 35001822 10.1080/10903127.2021.1977877

[8] Redlener M Olivieri P Loo GT et al . National assessment of quality programs in emergency medical services. Prehosp Emerg Care. 2018;22(3):370–8. 29297735 10.1080/10903127.2017.1380094

[9] Delbridge TR Bailey B Chew JL Jr. et al . EMS agenda for the future: where we are…where we want to be. Prehosp Emerg Care. 1998;2(1):1–12. 9737400 10.1080/10903129808958832

[10] Chahine S Kulasegaram KM Wright S et al . A call to investigate the relationship between education and health outcomes using big data. Acad Med. 2018;93(6):829–32. 29538109 10.1097/ACM.0000000000002217

[11] Vithalani V Sondheim S Cornelius A et al . Quality management of prehospital airway programs: an NAEMSP position statement and resource document. Prehop Emerg Care. 2022;26(sup1):14–22. 10.1080/10903127.2021.1989530 35001828

[12] Page, Wolfberg & Wirth . An imaginary barrier: how HIPAA promotes bidirectional patient data exchange with emergency medical services. 2019. Available at: https://nemsis.org/wp-content/uploads/2020/07/HIPAA_An-Imaginary-Barrier-to-Data-Exchange.pdf. Accessed July 21, 2024.

[13] Morrison L Cassidy L Welsford M et al . Clinical performance feedback to paramedics: what they receive and what they need. AEM Educ Train. 2017;1(2):87–97. 30051016 10.1002/aet2.10028 PMC6001722

[14] Eaton-Williams P Mold F Magnusson C . Exploring paramedic perceptions of feedback using a phenomenological approach. Br Paramed J. 2020;5(1):7–14. 33456380 10.29045/14784726.2020.06.5.1.7 PMC7783907

[15] O’Hara R Johnson M Siriwardena AN et al . A qualitative study of systemic influences on paramedic decision making: care transitions and patient safety. J Health Serv Res Policy. 2015;20(1 Suppl):45–53. 10.1177/1355819614558472 25472989

[16] Campbell EG Rosenthal M . Reform of continuing medical education: investments in physician human capital. JAMA. 2009;302(16):1807–8. 19861675 10.1001/jama.2009.1560

[17] Wolters Kluwer . Continuing medical education with UpToDate. 2024. Available at: https://www.wolterskluwer.com/en/solutions/uptodate/about/continuing-medical-education. Accessed July 21, 2024.

[18] Cate OT Carraccio C . Envisioning a true continuum of competency-based medical education, training, and practice. Acad Med. 2019;94(9):1283–8. 31460916 10.1097/ACM.0000000000002687

[19] Zafar MA Diers T Schauer DP et al . Connecting resident education to patient outcomes: the evolution of a quality improvement curriculum in an internal medicine residency. Acad Med. 2014;89(10):1341–7. 25054419 10.1097/ACM.0000000000000424

[20] Klein D Staples J Pittman C et al . Using electronic clinical practice audits as needs assessment to produce effective continuing medical education programming. Med Teach. 2012;34(2):151–4. 22288993 10.3109/0142159X.2012.644826

[21] Lammers RL Willoughby-Byrwa MJ Vos DG et al . Comparison of four methods of paramedic continuing education in the management of pediatric emergencies. Prehosp Emerg Care. 2022;26(4):463–75. 33872104 10.1080/10903127.2021.1916140

[22] Gugiu MR McKenna KD Platt TE et al . A proposed theoretical framework for clinical judgment in EMS. Prehosp Emerg Care. 2023;27(4):427–31. 35244513 10.1080/10903127.2022.2048756

[23] Hubner P Lobmeyr E Wallmuller C et al . Improvements in the quality of advanced life support and patient outcome after implementation of a standardized real-life post-resuscitation feedback system. Resuscitation. 2017;120:38–44. 28864072 10.1016/j.resuscitation.2017.08.235

[24] Scholz KH Hilgers R Ahlersmann D et al . Contact-to-balloon time and door-to-balloon time after initiation of a formalized data feedback in patients with acute ST-elevation myocardial infarction. Am J Cardiol. 2008;101(1):46–52. 18157964 10.1016/j.amjcard.2007.07.078

[25] Samuel A Cervero RM Durning SJ et al . Effect of continuing professional development on health professionals’ performance and patient outcomes: a scoping review of knowledge syntheses. Acad Med. 2021;96(6):913–23. 33332905 10.1097/ACM.0000000000003899

[26] Commission on Accreditation for Pre-Hospital Continuing Education . About CAPCE. Available at: https://www.capce.org/Home/About. Accessed April 2024.

[27] Hsieh HF Shannon SE . Three approaches to qualitative content analysis. Qual Health Res. 2005;15(9):1277–88. 16204405 10.1177/1049732305276687

[28] McGuire S Luke A Klassen A et al . 11 What medics want: analysis of feedback and patient follow-up requests from out-of-hospital providers at an academic emergency department. Ann Emerg Med. 2021;78(4):S5.

[29] Pollard J Black S . Do paramedics find it benefecial to learn the diagnosis given to their patients in the emergency department? Emerg Med J. 2015;32(5):421–1.

[30] Torres AJ McCoy RG . How to better value EMS clinicians as key care team members. AMA J Ethics. 2022;24(9):E898–905. 36170424 10.1001/amajethics.2022.898 PMC9557067

[31] Cash RE Rivard MK Chrzan K et al . Comparison of volunteer and paid EMS professionals in the United States. Prehosp Emerg Care. 2021;25(2):205–12. 32271639 10.1080/10903127.2020.1752867

[32] Janssen A Coggins A Tadros J et al . Delivering adaptive online learning using electronic health data: a feasibility and acceptability study with emergency trainees. Res Sq. 2023. Available at: 10.21203/rs.3.rs-2395367/v1.

