# Supplementary material for: Perceptions and Use of Automated Hospital Outcome Data by EMS Providers: A Pilot Study
Source: West J Emerg Med. 2024 Oct 1;25(6):949–57. doi: 10.5811/westjem.21175 (PMC11610734; doi:10.5811/westjem.21175)

**Supplement Tables:**

Table S1: Presurvey and Post Survey Responses stratified by Certification Level

|  | **Paramedic** | | | | | **Other** | | | | |
| --- | --- | --- | --- | --- | --- | --- | --- | --- | --- | --- |
|  | **Pre** | | **Post** | |  | **Pre** | | **Post** | |  |
| % (n) | **Disagree** | **Agree** | **Disagree** | **Agree** | **p-value** | **Disagree** | **Agree** | **Disagree** | **Agree** | **p-value** |
| I review outcomes to improve the care I provide to patients. | 7.3% (12) | 92.7% (152) | 4.1% (5) | 95.9% (117) | 0.26 | 0.0% (0) | 100% (36) | 4% (1) | 96.0% (24) | _ |
| I review outcomes to improve my clinical knowledge. | 6.1% (10) | 93.9% (153) | 0.8% (1) | 99.2% (118) | 0.03 | 2.8% (1) | 97.2% (35) | 8.3% (2) | 91.7% (22) | 0.56 |
| I review outcomes to know whether my care made a difference. | 7.3% (12) | 92.7% (152) | 3.3% (4) | 96.7% (118) | 0.19 | 5.6% (2) | 94.4% (34) | 8.3% (2) | 91.70% | 0.99 |
| I review outcomes to know whether I provided the right care. | 6.6% (11) | 93.4% (155) | 2.5% (3) | 97.5% (118) | 0.16 | 8.3% (3) | 91.7% (33) | 4.2% (1) | 95.8% (23) | 0.64 |
| I review outcomes to obtain closure on patient encounters. | 14.6% (24) | 85.4% (140) | 9.0% (11) | 91.0% (111) | 0.15 | 11.1% (4) | 88.9% (32) | 8.3% (2) | 91.7% (22) | 0.99 |
| Reviewing hospital outcome data through the patient outcomes feature helps improve my job satisfaction. | 12.6% (21) | 87.4% (146) | 5.7% (7) | 94.3% (116) | 0.05 | 6.7% (3) | 93.3% (42) | 3.7% (1) | 96.3% (26) | 0.99 |
| Reviewing hospital outcome data through the patient outcomes feature helps improve my clinical knowledge. | 7.4% (12) | 92.6% (151) | 5.0% (6) | 95.0% (114) | 0.42 | 4.7% (2) | 95.4% (41) | 16.0% (4) | 84.00% | 0.18 |
| If I were provided 15 minutes of approved continuing education credit for each patient outcome I reviewed, I would be more likely to review my patient outcomes in the patient outcomes feature. | 6.7% (11) | 93.3% (154) | 7.3% (9) | 92.7% (115) | 0.84 | 13.3% (6) | 86.7% (39) | 19.2% (5) | 80.8% (21) | 0.52 |

Table S2: Presurvey and Post Survey Responses stratified by Years of EMS Experience

|  | Survey Time | # Cases | % Cases | Acquire Personal Knowledge | Enhance Personal Confidence and Competence | Improvement of Personal Clinical Practice | Operations | Support System-Wide Improvement in Clinical Quality | Unknown |
| --- | --- | --- | --- | --- | --- | --- | --- | --- | --- |
| All respondents | Pre | 57 | 100% | 32% | 23% | 23% | 11% | 9% | 3% |
|  | Post | 29 | 100% | 14% | 29% | 21% | 19% | 10% | 7% |
| Paramedic | Pre | 42 | 74% | 32% | 24% | 27% | 9% | 6% | 3% |
|  | Post | 21 | 72% | 13% | 29% | 26% | 16% | 13% | 3% |
| EMT | Pre | 10 | 18% | 47% | 27% | 7% | 0% | 13% | 7% |
|  | Post | 3 | 10% | 33% | 50% | 17% | 0% | 0% | 0% |
| Non-certified & other | Pre | 5 | 9% | 0% | 0% | 14% | 57% | 29% | 0% |
|  | Post | 4 | 14% | 0% | 0% | 0% | 75% | 0% | 25% |
| Experience  0-4 years | Pre | 13 | 23% | 40% | 20% | 15% | 15% | 10% | 0% |
|  | Post | 6 | 21% | 33% | 22% | 11% | 33% | 0% | 0% |
| Experience  5-10 years | Pre | 10 | 18% | 32% | 39% | 18% | 7% | 4% | 0% |
|  | Post | 5 | 17% | 13% | 25% | 38% | 13% | 13% | 0% |
| Experience  11-20 years | Pre | 23 | 40% | 22% | 19% | 35% | 11% | 8% | 5% |
|  | Post | 10 | 34% | 15% | 38% | 23% | 8% | 8% | 8% |
| Experience  21 years and greater | Pre | 11 | 19% | 44% | 6% | 13% | 13% | 19% | 6% |
|  | Post | 7 | 24% | 0% | 27% | 18% | 27% | 18% | 9% |

Table S3: Presurvey and Post Survey Responses stratified by Role

|  | **Patient Care Provider** | | | | | **Other** | | | | |
| --- | --- | --- | --- | --- | --- | --- | --- | --- | --- | --- |
|  | **Pre** | | **Post** | |  | **Pre** | | **Post** | |  |
| % (n) | **Disagree** | **Agree** | **Disagree** | **Agree** | **p-value** | **Disagree** | **Agree** | **Disagree** | **Agree** | **p-value** |
| I review outcomes to improve the care I provide to patients. | 1.6% (2) | 98.4% (125) | 2.0% (2) | 98.0% (100) | 0.99 | 13.7% (10) | 86.3% (63) | 8.9% (4) | 91.1% (41) | 0.56 |
| I review outcomes to improve my clinical knowledge. | 2.4% (3) | 97.6% (123) | 1.0% (1) | 99.0% (98) | 0.63 | 11.0% (8) | 89.0% (65) | 4.5% (2) | 95.5% (42) | 0.32 |
| I review outcomes to know whether my care made a difference. | 2.4% (3) | 97.6% (123) | 2.0% (2) | 98.0% (100) | 0.99 | 14.9% (11) | 85.1% (63) | 9.1% (4) | 90.9% (40) | 0.41 |
| I review outcomes to know whether I provided the right care. | 3.2% (4) | 96.9% (123) | 2.0% (2) | 98.0% (98) | 0.70 | 13.3% (10) | 86.7% (65) | 4.4% (2) | 95.6% (43) | 0.21 |
| I review outcomes to obtain closure on patient encounters. | 10.3% (13) | 89.7% (113) | 6.9% (7) | 93.1% (94) | 0.34 | 20.3% (15) | 79.7% (59) | 13.3% (6) | 86.7% (39) | 0.37 |
| Reviewing hospital outcome data through the patient outcomes feature helps improve my job satisfaction. | 8.4% (11) | 91.6% (120) | 3.0% (3) | 97.0% (98) | 0.10 | 16.1% (13) | 83.9% (68) | 10.4% (5) | 89.6% (43) | 0.37 |
| Reviewing hospital outcome data through the patient outcomes feature helps improve my clinical knowledge. | 1.6% (2) | 98.4% (124) | 4.0% (4) | 96.0% (96) | 0.41 | 15.0% (12) | 85.0% (68) | 13.6% (6) | 86.4% (38) | 0.84 |
| If I were provided 15 minutes of approved continuing education credit for each patient outcome I reviewed, I would be more likely to review my patient outcomes in the patient outcomes feature. | 4.6% (6) | 95.4% (124) | 7.8% (8) | 92.2% (94) | 0.31 | 13.8% (11) | 86.3% (69) | 10.6% (5) | 89.4% (42) | 0.61 |

Table S4: Presurvey and Post Survey Responses stratified by Historical Frequency of Outcomes Review

|  | **Frequently** | | | | | **Occasionally/Rarely** | | | | |
| --- | --- | --- | --- | --- | --- | --- | --- | --- | --- | --- |
|  | **Pre** | | **Post** | |  | **Pre** | | **Post** | |  |
| % (n) | **Disagree** | **Agree** | **Disagree** | **Agree** | **p-value** | **Disagree** | **Agree** | **Disagree** | **Agree** | **p-value** |
| I review outcomes to improve the care I provide to patients. | 3.3% (5) | 96.7% (145) | 1.9% (2) | 98.1% (105) | 0.70 | 14.0% (7) | 86.0% (43) | 10.0% (4) | 90.0% (36) | 0.75 |
| I review outcomes to improve my clinical knowledge. | 2.7% (4) | 97.3% (144) | 1.0% (1) | 99.0% (103) | 0.65 | 13.7% (7) | 86.3% (44) | 5.1% (2) | 94.9% (29) | 0.29 |
| I review outcomes to know whether my care made a difference. | 4.7% (7) | 95.3% (142) | 1.9% (2) | 98.1% (104) | 0.31 | 13.7% (7) | 86.3% (44) | 10.0% (4) | 90.0% (36) | 0.75 |
| I review outcomes to know whether I provided the right care. | 4.0% (6) | 96.0% (144) | 1.9% (2) | 98.1% (102) | 0.48 | 15.4% (8) | 84.6% (44) | 4.9% (2) | 95.1% (39) | 0.18 |
| I review outcomes to obtain closure on patient encounters. | 10.1% (15) | 89.9% (134) | 1.9% (2) | 98.1% (103) | 0.01 | 25.5% (13) | 74.5% (38) | 26.8% (11) | 73.2% (20) | 0.99 |
| Reviewing hospital outcome data through the patient outcomes feature helps improve my job satisfaction. | 7.9% (12) | 92.1% (140) | 0.9% (1) | 99.1% (106) | 0.02 | 20.0% (12) | 80.0% (48) | 16.3% (7) | 83.7% (36) | 0.63 |
| Reviewing hospital outcome data through the patient outcomes feature helps improve my clinical knowledge. | 4.8% (7) | 95.2% (140) | 1.9% (2) | 98.1% (101) | 0.31 | 11.9% (7) | 88.1% (52) | 19.1% (8) | 80.9% (34) | 0.32 |
| If I were provided 15 minutes of approved continuing education credit for each patient outcome I reviewed, I would be more likely to review my patient outcomes in the patient outcomes feature. | 6.0% (9) | 94.0% (141) | 8.5% (9) | 91.5% (97) | 0.44 | 13.3% (8) | 86.7% (52) | 11.4% (5) | 88.6% (39) | 0.76 |

**Supplemental Figures:**

Figure S1: Initial Codes and Themes


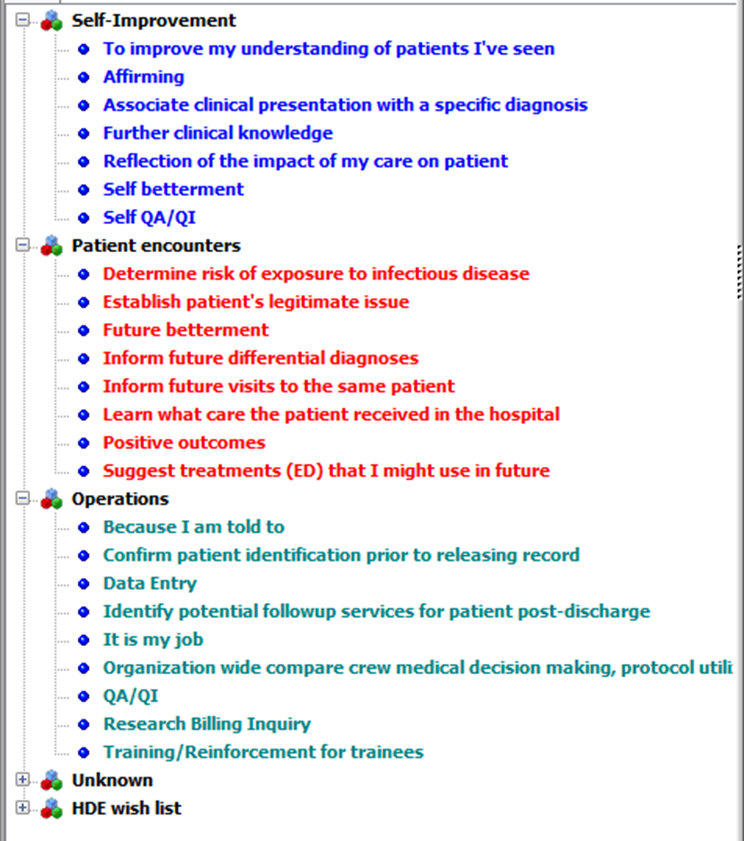


Figure S2: Final Codes and Themes


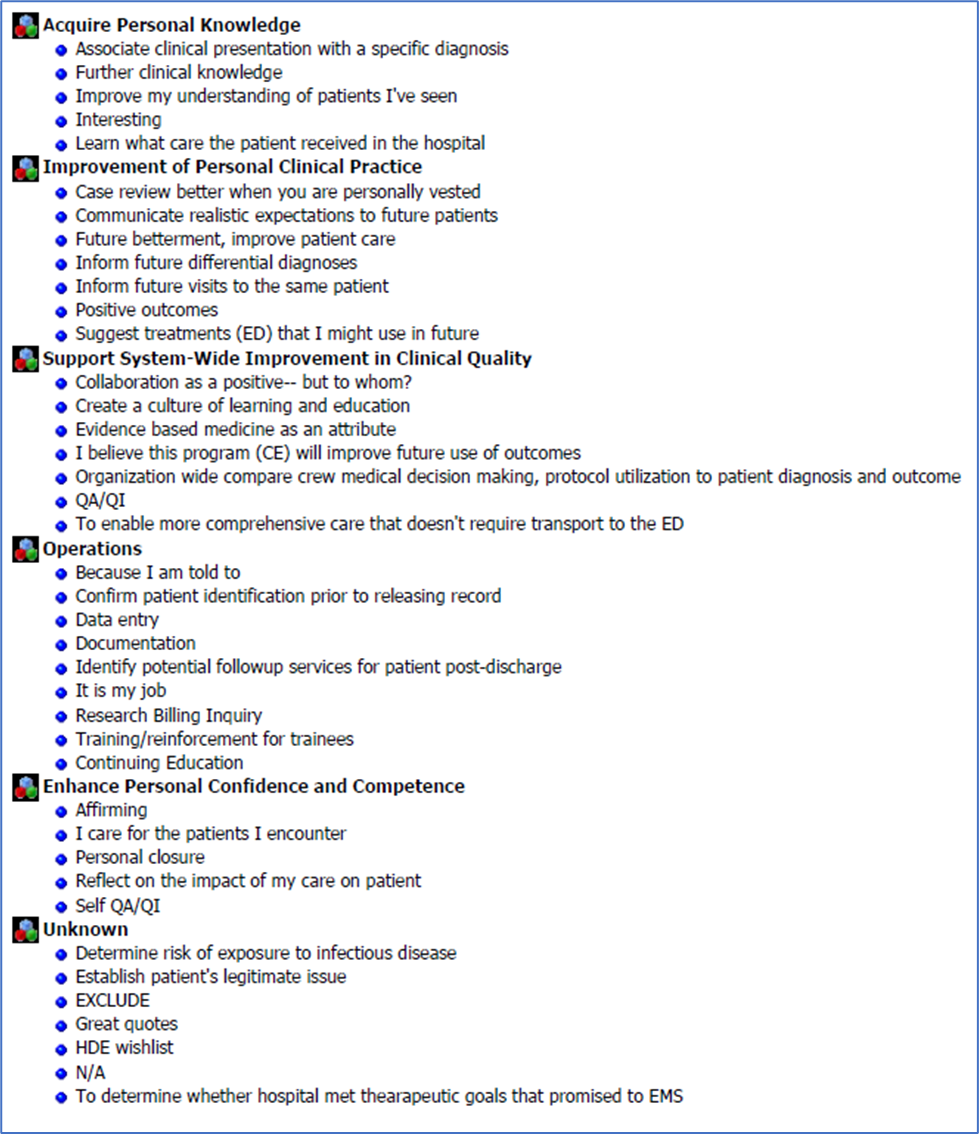

Supplement: Supplementary file 1 [file wjem-25-949-s001.docx]
